# Supplementary material for: Modeling glioblastoma heterogeneity as a dynamic network of cell states
Source: Mol Syst Biol. 2021 Sep 16;17(9):e10105. doi: 10.15252/msb.202010105 (PMC8444284; doi:10.15252/msb.202010105)
Supplement: Supplementary file 6 — Source Data for Figure 5 [file MSB-17-e10105-s004.zip › Figure5A_sourcedata/GSEA_3017/hallmarks_stateD.GseaPreranked.1621934542601/HALLMARK_EPITHELIAL_MESENCHYMAL_TRANSITION.html]

Details for gene set HALLMARK\_EPITHELIAL\_MESENCHYMAL\_TRANSITION[GSEA]

|  || Dataset | state23017 |
| Phenotype | NoPhenotypeAvailable |
| Upregulated in class | na\_neg |
| GeneSet | HALLMARK\_EPITHELIAL\_MESENCHYMAL\_TRANSITION |
| Enrichment Score (ES) | -0.42966995 |
| Normalized Enrichment Score (NES) | -1.9297147 |
| Nominal p-value | 0.0022050717 |
| FDR q-value | 0.02930139 |
| FWER p-Value | 0.103 |
Table: GSEA Results Summary

  

Fig 1: Enrichment plot: HALLMARK\_EPITHELIAL\_MESENCHYMAL\_TRANSITION      
 Profile of the Running ES Score & Positions of GeneSet Members on the Rank Ordered List

  

| PROBE | GENE SYMBOL | GENE\_TITLE | RANK IN GENE LIST | RANK METRIC SCORE | RUNNING ES | CORE ENRICHMENT || 1 | VIM |  |  | 75 | 0.314 | -0.0399 | No |
| 2 | LGALS1 |  |  | 83 | 0.308 | -0.0271 | No |
| 3 | ITGB1 |  |  | 147 | 0.260 | -0.0608 | No |
| 4 | GPC1 |  |  | 213 | -0.255 | -0.0964 | No |
| 5 | ITGAV |  |  | 217 | -0.255 | -0.0836 | No |
| 6 | TPM2 |  |  | 229 | -0.257 | -0.0769 | No |
| 7 | FSTL1 |  |  | 278 | -0.261 | -0.0988 | No |
| 8 | FGF2 |  |  | 287 | -0.263 | -0.0895 | No |
| 9 | SDC1 |  |  | 300 | -0.264 | -0.0832 | No |
| 10 | FLNA |  |  | 338 | -0.267 | -0.0962 | No |
| 11 | FBN2 |  |  | 401 | -0.273 | -0.1284 | No |
| 12 | GJA1 |  |  | 453 | -0.280 | -0.1515 | No |
| 13 | EFEMP2 |  |  | 503 | -0.285 | -0.1728 | No |
| 14 | CD59 |  |  | 622 | -0.304 | -0.2468 | No |
| 15 | DPYSL3 |  |  | 722 | -0.323 | -0.3048 | No |
| 16 | TIMP3 |  |  | 784 | -0.339 | -0.3322 | No |
| 17 | MEST |  |  | 864 | -0.360 | -0.3725 | No |
| 18 | PMEPA1 |  |  | 868 | -0.363 | -0.3533 | No |
| 19 | THY1 |  |  | 895 | -0.372 | -0.3515 | No |
| 20 | DST |  |  | 913 | -0.379 | -0.3423 | No |
| 21 | ELN |  |  | 924 | -0.384 | -0.3274 | No |
| 22 | CDH2 |  |  | 1007 | -0.418 | -0.3665 | No |
| 23 | PRRX1 |  |  | 1089 | -0.464 | -0.4022 | Yes |
| 24 | NTM |  |  | 1094 | -0.466 | -0.3776 | Yes |
| 25 | CADM1 |  |  | 1103 | -0.469 | -0.3561 | Yes |
| 26 | SAT1 |  |  | 1109 | -0.473 | -0.3319 | Yes |
| 27 | SPARC |  |  | 1127 | -0.493 | -0.3160 | Yes |
| 28 | CDH6 |  |  | 1193 | -0.574 | -0.3326 | Yes |
| 29 | ITGB5 |  |  | 1205 | -0.590 | -0.3063 | Yes |
| 30 | PLOD2 |  |  | 1215 | -0.615 | -0.2768 | Yes |
| 31 | SERPINE1 |  |  | 1218 | -0.617 | -0.2418 | Yes |
| 32 | COL5A2 |  |  | 1236 | -0.659 | -0.2159 | Yes |
| 33 | PTX3 |  |  | 1242 | -0.679 | -0.1796 | Yes |
| 34 | APLP1 |  |  | 1249 | -0.700 | -0.1428 | Yes |
| 35 | MMP2 |  |  | 1260 | -0.733 | -0.1072 | Yes |
| 36 | CD44 |  |  | 1270 | -0.762 | -0.0691 | Yes |
| 37 | SPP1 |  |  | 1277 | -0.787 | -0.0271 | Yes |
| 38 | PMP22 |  |  | 1302 | -1.010 | 0.0140 | Yes |
Table: GSEA details [plain text format]

  

Fig 2: HALLMARK\_EPITHELIAL\_MESENCHYMAL\_TRANSITION: Random ES distribution      
 Gene set null distribution of ES for **HALLMARK\_EPITHELIAL\_MESENCHYMAL\_TRANSITION**
